# How Do Teleworkers and Organizations Manage the COVID-19 Crisis in Brazil? The Role of Flexibility I-Deals and Work Recovery in Maintaining Sustainable Well-Being at Work

**DOI:** 10.3390/ijerph182312522

**Published:** 2021-11-28

**Authors:** Felisa Latorre, Amalia Raquel Pérez-Nebra, Fabiana Queiroga, Carlos-María Alcover

**Affiliations:** 1 Department of Psychology, Faculty of Health Sciences, Universidad Rey Juan Carlos, Avda. Atenas s/n, 28922 Alcorcón, Spain; felisa.latorre@urjc.es (F.L.); carlosmaria.alcover@urjc.es (C.-M.A.); 2 Department of Management, Campus Universitário Darcy Ribeiro, Universidade de Brasília, Brasilia 70910-900, Brazil; 3 Department of Psychology, Universidad Internacional de Valencia, 46002 Valencia, Spain; 4 Department of Psychology, Université Côte D’Azur, Avenue des Diables Bleus, 06357 Nice, France; fabiana.queiroga@univ-cotedazur.fr

**Keywords:** COVID-19, recovery from work stress, sustainable well-being at work, teleworkers, idiosyncratic deals (i-deals), performance, coping strategies

## Abstract

The COVID-19 pandemic has impacted the economic market and labor contexts worldwide. Brazil has suffered one of the worst social and governmental managements of the COVID-19 crisis, forcing workers and organizations to develop coping strategies. This environment can affect both well-being and performance at work. Sustainable well-being at work refers to different patterns of relationships between performance and well-being. It may include eudaimonic (e.g., Meaning of Work—MOW) or hedonic (e.g., emotions) forms of well-being. This study tests the moderating role of recovery from work stress in the relationship between flexibility i-deals and patterns of sustainable well-being at work in Brazilian teleworkers. We relied on two studies to achieve this objective. In Study 1, conducted during the pandemic’s first outbreak in Brazil (*N* = 386), recovery experiences moderated the relationship between i-deals and clusters formed by performance and MOW (eudaimonic happiness). In Study 2, conducted during the second outbreak (*N* = 281), we identified relationships between clusters of emotions (hedonic happiness) and MOW (eudaimonic) with performance. The results supported the idea that recovery experiences moderated the relationship between i-deals and patterns of sustainable well-being at work differently. Our findings have implications for Human Resource Management and teleworkers, especially for employee behaviors to deal with stress.

## 1. Introduction

The 2021 outbreak of the COVID-19 pandemic has caused more than five million deaths worldwide [1]. These are accompanied by a disruption of every aspect of life, including economic and financial threats, job insecurity, and physical and mental health challenges such as stress and anxiety. Governments have tried to reduce community transmission using strong control policies [2]. More than 21,000,000 COVID-19 cases have been confirmed in Brazil, making it the country with the third most confirmed COVID-19 cases [1]. More than 607,000 deaths have been reported, and the number is still growing. Aside from COVID cases, Brazilians have experienced the pandemic harshly [3]. Stress has impacted their well-being and performance differently [4], depending on their coping abilities and how they deal with norms [5] and stress.

Amid this pandemic uncertainty in Brazil [6], organizations and teleworkers have developed strategies to cope with the problems, attempting to enhance or preserve their labor and personal resources. Brazilians show low trust in institutions [7] and a collectivist and loose [5,8,9] culture. Consequently, interpersonal relationships and individual experiences are especially salient characteristics to predict individual outcomes [9]. Thus, organizations and teleworkers have established idiosyncratic deals (i-deals), defined as “special conditions of employment negotiated between an individual worker and his or her employer” [10] (p. 7). In addition, teleworkers have developed personal strategies for off-job recovery from work stress, engaging in activities that lead to psychological detachment, relaxation, mastery, and control [11,12]. Cognitive, emotional, and physical detachments after work are crucial predictors for employees’ health [13]. These special conditions lead to increased well-being and performance [14,15].

The Happy-Productive Worker Thesis (HPWT) suggests that well-being and performance are related, but other researchers have shown that well-being and performance are not always aligned. This new proposition has been called Sustainable Well-Being at Work (SWBW) [16], and it expands the study of the HPWT to cases where the former thesis does not fit. The SWBW proposal includes four relationship patterns between well-being and performance [17,18], creating four types of workers: (1) unhappy and unproductive, (2) happy and unproductive, (3) unhappy and productive, and (4) happy and productive [16,17,18]. The SWBW states that the happy-productive group is the most sustainable for the organization and the worker.

Previous studies have analyzed the relationship between i-deals and performance [19] or well-being [20] without examining their synergic interactions or the SWBW patterns. Additionally, recovery has been related to well-being [21] or performance [22], but the synergy between well-being and performance and its moderating role were not considered.

Hence, we tested the moderating role of recovery from work stress in the relationship between flexibility i-deals and patterns of SWBW in Brazilian teleworkers at two time points during the COVID-19 pandemic, in 2020 and 2021. Moreover, we tested the SWBW patterns in a previously ignored international sample, a Latin American population. This study expands the existing body of knowledge regarding SWBW by providing new evidence of the relationship between personal and labor resources and describing how this relationship could promote sustainable well-being at work.

### 1.1. Theoretical Background and Hypothesis

Over the last 20 months, Brazilians have been highly distressed. As the Job Demands and Control model [4,23] has described, low job control and a high psychological demand create poorly motivated high-strain workers. Organizations and employees have tried to diminish the lack of control, providing i-deals or developing strategies to experience recovery to reduce the negative impact on personal and organizational outcomes. Combining organizational (i.e., i-deals) with personal (i.e., work stress recovery) strategies should promote the sustainable pattern (happy-productive).

#### 1.1.1. Sustainable Well-Being at Work (SWBW)

The global pandemic situation has been difficult; however, the situation in Brazil has been far worse [5]. In this environment, organizations and teleworkers have aspired to develop sustainable positive relations to maintain the synergy between well-being and performance necessary for individual and organizational outputs that lead to high-performance teleworkers.

The well-being and the performance of workers have long been studied in management research and practice [24]. In summary, happy workers perform more efficiently than non-happy workers [24,25]; this is called the Happy and Productive Worker Thesis (HPWT). However, most studies have only published positive relations and neglected negative or null relations [18]. The sustainable well-being at work (SWBW) thesis, proposed by Peiró et al. [16], emerged from HPWT [25] and expanded it in several ways [16,26]. First, it considers that the relationship between well-being and performance is more complex than a linear relation. Second, this relation is perhaps measure-dependent. Well-being is measured mainly by hedonic well-being (e.g., happiness, positive emotions, job satisfaction); however, other operationalizations can be used, for example, eudaimonic well-being (e.g., Meaning of Work) [26]. Third, they suggest that both constructs are longitudinally synergistic, which can have two interpretations: (1) well-being and job performance show an enduring symbiosis, or (2) they are mutually reinforcing, contributing to an upward spiral of well-being and performance.

**Figure 1:**
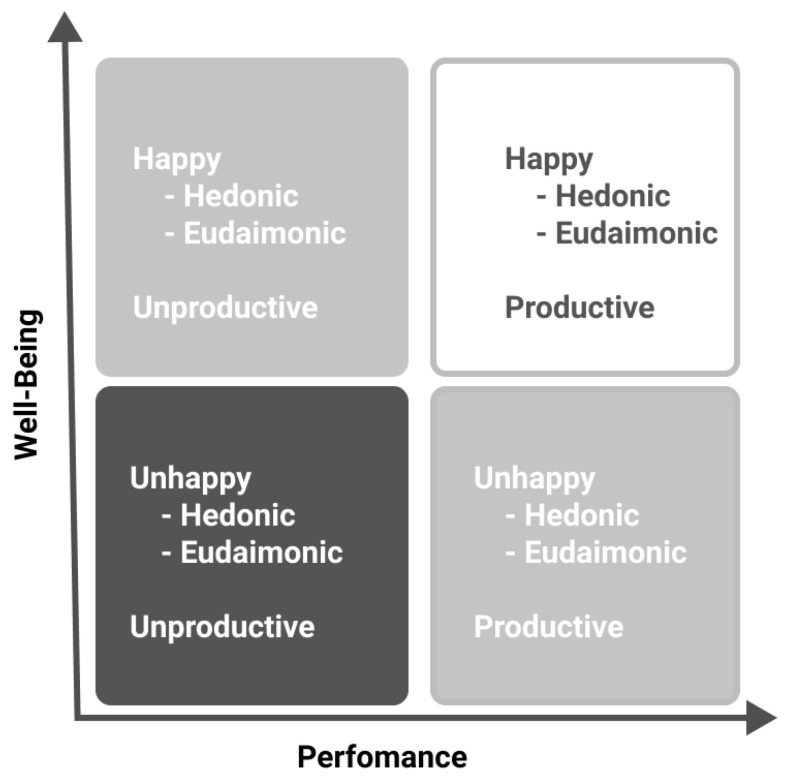
Sustainable well-being at work [16].

The SWBW identified four patterns considering worker behaviors: happy-productive, happy-unproductive, unhappy-productive, and unhappy-unproductive (Figure 1). Previous results under the pandemic context supported these four patterns [17,18]. Because SWBW derives from HPWT, we will use happiness as a synonym of well-being. In addition, we expect workers to fluctuate between those patterns over time. For instance, when a PhD student is writing a paper, he or she will feel unhappy but productive for a period until the paper is published, and he or she feels both happy and productive. The problem arises when a misaligned pattern continues for a longer-term. In this sense, the happy and productive pattern is considered more sustainable than the other patterns of well-being at work.

Besides identifying the four patterns, the SWBW showed that those patterns could be estimated using different well-being (eudaimonic or hedonic) and performance operationalizations [17,18]. The first pandemic outbreak differentiated those considered “essential” workers from “the rest”. Therefore, this crisis exposed people to a fundamental question: the Meaning of Work (MOW) [27]. MOW is defined as the amount of significance the work has for an individual [28] and the extent to which workers perceive their work as relevant and conducive to self-development. MOW is a future-oriented eudaimonic construct [26]; eudaimonic well-being or eudaimonic happiness refers to the “ideal in the sense of excellence giving meaning and direction to life and is necessary to more fully understand the relationship between happiness and productivity” [26] (p. 1).

In contrast to eudaimonic well-being, hedonic well-being could be defined as attaining pleasure and avoiding pain [29]. This term has frequently been split into affective and subjective well-being. Affective well-being has been operationalized as a broad construct extending to the whole life (e.g., mental health) and as a medium-range construct referring to a specific segment of life (e.g., job satisfaction [30]). However, subjective well-being refers to people’s cognitive and emotional assessment of their lives (e.g., life satisfaction [31]). Some research has related flexibility i-deals with hedonic happiness (see [14] as an example).

According to Gallup’s report [2], the eudaimonic and hedonic well-being dimensions declined during the pandemic in Brazil, contrasting with other countries that improved their well-being. The report reveals that flexible work schedules and supportive management have become even more critical (components of i-deals definitions) to teleworkers. Previous research has shown that the time employees work from home is directly associated with the time they spend contacting their supervisors [32] and developing i-deals [33]. Since the pandemic crisis started in Brazil, many workers have reported work overload and renegotiated previous conditions, e.g., [34], emphasizing the need for efficient communication between managers and employees. Thus, flexibility i-deals seem to be a relevant driver for employees that could improve sustainable patterns of well-being and performance at work.

#### 1.1.2. Idiosyncratic Deals (i-Deals)

Maintaining organizations’ outputs in many sectors (e.g., energy, food, education) is necessary to preserve life, public safety, and basic societal functioning. Brazilian organizations and employees developed individual conditions for teleworkers, attempting to maintain their outputs via i-deals. These are voluntary agreements between the employer and the employee [10] that, according to a meta-analysis [35], benefit both. I-deals also benefit organizations because they allow employees to obtain arrangements that can motivate and accommodate their unique situations [20], thus increasing or maintaining performance [19,36]. Although i-deals seem to contribute to productivity [33] and enhanced client satisfaction [14], this relationship is only partially supported [19].

I-deals benefit the employee by reducing their emotional exhaustion and increasing their performance, measured as client satisfaction [14]. In particular, i-deals benefit knowledge employees because their performance depends on cognitive processes that can be affected by negative emotions, which may be especially relevant in the stressful context of COVID-19. I-deals enable employees to face role stress, workload, and work pressure [36]. They might also contribute to the development of positive affect [37]; for instance, empirical research found that i-deals increased employee job satisfaction [38].

The three most common forms of i-deals are developmental, flexible, and workload reduction [39]. For this work, we will focus on flexibility i-deals, which are related to the work schedule. This type of i-deal is prevalent in organizations and has a strong potential to influence employees [40]. Due to the fast-paced pandemic and the home invasion of their job tasks, flexibility i-deals must be handled to help workers perform better in their work and private lives [19] and to increase well-being. Flexibility i-deals are likely to provide a better work-life balance, bringing more personal energies to their different roles [41]. The flexibility i-deals provide workers with the opportunity to fit with their jobs, broaden their action repertoires, become more focused, and support others [20]. On this basis, flexibility i-deals could be considered a resource to cope with the stressful pandemic environment in Brazil, helping to develop and maintain high levels of performance and well-being, in other words, fostering sustainable well-being at work [42]. Supportive management and job flexibility (e.g., flexible work schedules) have been important antecedents of well-being during the pandemic [2]. Workers value how and when to work more than ever. There is some evidence that flexibility i-deals have been established by some teleworkers in the pandemic environment in Brazil [43].

I-deals represent the Human Resource Management system [36] that questions the “one-size-fits-all” approach. The pandemic is a perfect motivation for managers to implement them, considering the need for accommodation to regulate job content and conditions [44]. Although flexibility i-deals present inconclusive results regarding performance [36], the results came from a different context (German-individualistic [45], non-pandemic with issues in work-family balance) that cannot be generalized. Flexibility i-deals diminish stress (e.g., emotional exhaustion [14]) and improve well-being and performance because teleworkers can manage their personal and job demands. Thus, flexibility i-deals have the potential to improve SWBW during the Brazilian pandemic environment.

Hence, we hypothesized that:

**Hypothesis** **1** **(H1).** *High levels of flexibility i-deals will be related to sustainable patterns of well-being and performance.*

#### 1.1.3. Recovery as a Worker Moderating Resource

Recovery from work is a process whereby individual functional systems aquired during a stressful experience return to their pre-stressor levels [27]. During the pandemic outbreak, having the opportunity to recover from work strain was a critical personal resource that could help employees be healthy and efficient [22]. Recovery as a psychological construct has been operationalized [11] into four recovery experiences: psychological detachment from work, relaxation, mastery, and control. Recovery is elicited by the rehabilitation process, a specific subjective experience in leisure time or sleeping [13]. It does not refer to any specific strategy; each person will experience a different psychological process through a specific strategy. Thus, psychological detachment-oriented activities imply leaving one’s work behind in psychological terms (e.g., physical exercise or social relationships); the relaxation-oriented experiences are often associated with activities aimed at relaxation (e.g., going for a walk or reading a book); mastery-oriented activities support the recovery process by increasing new resources such as challenging and learning opportunities in other domains aside from work (e.g., learning a new language); and control-oriented activities are related to the ability to choose an action from a range of options (e.g., choosing leisure experiences) [12,13]. Recovery could be conceptualized as the opposite of the strain process [46]. Empirical research has shown that recovery experiences diminish stress [47,48,49], increase well-being (e.g., [21]), and prevent burnout [50], except for psychological detachment, which is related to higher stress and burnout [49]. Therefore, recovery is sufficiently sustained to be considered a factor that reduces psychological and physiological stress and increases performance indicators [12,47,48].

The reduction of well-being and performance can be described as a lack of individual resources that may be reestablished during recovery [12,21]. In this line, Conservation of Resources Theory (COR, [51]) assumed that people struggle to obtain, retain, and protect their resources. Resources can be external entities such as objects or financial assets (i.e., houses, cars, clothes, food) or internal attributes such as personal characteristics or energies. Stress threatens these internal resources and may consequently harm health and well-being. The COR theory helped explain the relevance of recovery from stress in the Brazilian pandemic context, just as it had in other contexts (e.g., [52,53]), because keeping personal resources may moderate the relationship between labor resources (i-deals) and sustainable patterns, catalyzing the relationship.

Thus, we hypothesized that:

**Hypothesis** **2** **(H2).** *High levels of recovery experiences will moderate (by increasing) the relationship between flexibility i-deals and sustainable patterns of well-being and performance at work.*

**Figure 2:**
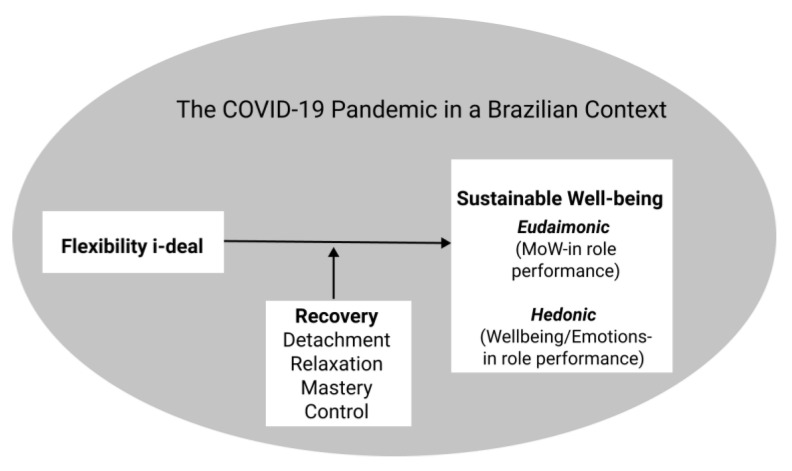
Theoretical model.

Figure 2 shows the theoretical model used in our study.

## 2. Study Context Overview

**Figure 3:**
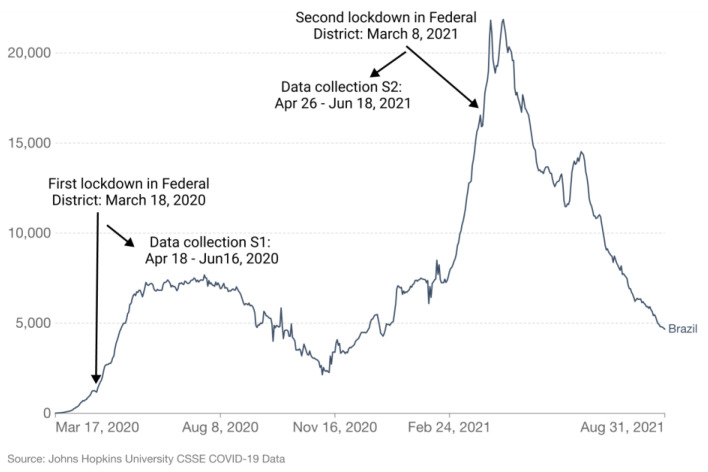
Weekly confirmed COVID-19 deaths in Brazil [56]. S1 = Study 1; S2 = Study 2.

Brazil has hit a worldwide tragic milestone regarding confirmed cases of and deaths from COVID-19 [54]. Some of the causes for this heartbreaking situation were the denialist mentality of the government, because the highest political level was skeptical about the gravity of the virus [6]; the lack of worldwide compulsory measures to avoid community transmission; confusing positions among governments (see Figure 3); the low level of trust (0.11) in public institutions [2]; a looser culture [5]; and income inequality that increases the risk of death [55], among other things.

**Figure 4:**
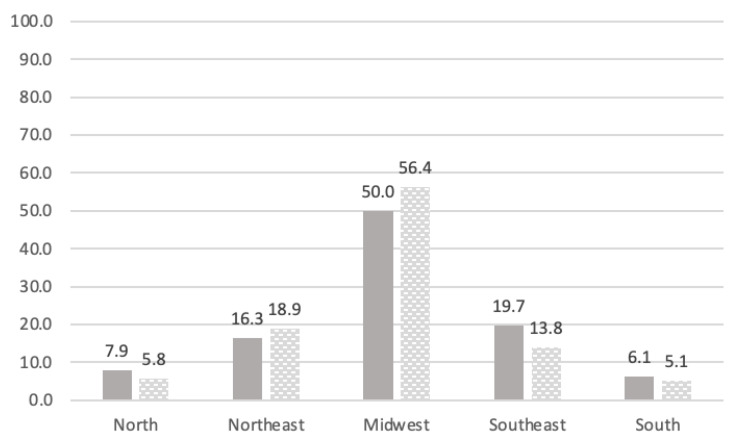
Percentage of respondents according to Brazilian region (Study 1, *N* = 386; Study 2, *N* = 281).

Faced with the Federal government’s denial, each Brazilian state adopted individual programs to contain the pandemic. The dates of confinement, when applied, varied considerably. The Federal District (FD), residence to most of this study’s survey respondents, was one of the first to declare lockdown. The dates presented in Figure 3 are based on the FD resolutions, which illustrate the period of each study, in the first and second COVID-19 outbreak in Brazil. Figure 4 presents the number of respondents per region.

Moreover, the contextual effect [55] could also justify the number of Brazilian cases and deaths during the pandemic and the extra stress on the population at all levels. Such conditions deteriorate the quality of life in Brazil. Other indicators of workers’ well-being also decreased from 2017 to 2020 [2], partly owing to the pandemic, but mainly due to job retention and low support from the government.

Additionally, the privileged workers who remained in their jobs and became teleworkers had to manage uncertainties regarding their jobs, tasks, health, and families’ health, so they were also affected. As reported in the media, many people lost their jobs, and those who remained had to handle isolation or work–family conflicts, sometimes in overcrowded homes [2]. The high level of uncertainty in Brazil because of the government’s denial of the pandemic [6] could have damaged mental health consequences [2,57].

According to COR theory [51,58], teleworkers could gain new resources, restore threatened resources, or lose resources. On the one hand, having work demands and facing stressful events leads to a stress spiral that may result in burnout, which has many consequences for work outputs and health [59]. On the other hand, increasing internal resources such as energy (physical exercise [60], self-efficacy employing experiences of mastery and control [61], or positive mood [62]) will additionally help to restore threatened resources [63]. This long-term perspective was the reason to conduct this research at the two peak times of outbreak death cases in Brazil; we tested the same model in both.

Hereafter, we will present the design of the studies conducted in the first and second outbreaks in Brazil. In the first outbreak, we measured only one type of well-being: the eudaimonic well-being based on the moment’s zeitgeist. In the second outbreak, we included a hedonic variable, inspired by the Gallup report [2] emotions.

## *3.* Study 1: Examining the Moderating Role of Recovery in the Relationship between I-Deals and Patterns of SWBW to Teleworkers in the First Brazilian Outbreak

### 3.1. Participants

The single inclusion criterion was to be teleworking due to pandemic containment measures. An online, social networking, non-probabilistic snowball sampling process recruited the sample because the study was conducted in the pandemic lockdown. Five-hundred forty respondents answered the survey, but only those with a formal employment relationship were retained. Thus, 386 employees were considered (47.7 fixed employees and 52.3 public servants), 62.2% women. Regarding marital status, most participants reported being married (61.4%) and 26.7% single. In terms of education level, 71.8% had a Master’s degree or higher, and 26.7% had graduated. With the high level of education, income was equally high, meaning that 55.2% earned more than 10 times the Brazilian minimum salary (The Brazilian minimum salary is equivalent to approximately USD 190.0). Concerning the occupational role, 49% indicated being subordinate, and 46.6% shared roles, that is, they were both boss and subordinate. Only 4.4% of the respondents were exclusively bosses.

Regarding experience in telework, 50.8% indicated that they had never worked remotely, and 39.4% said they “sometimes” worked remotely. Finally, we asked questions to understand the household population density within the teleworking environment. Respondents commonly shared a house with three other people (22.0%), lived with two people (25.4%), or at least one more person (23.3%). Additionally, 57.8% had at least one child living at home.

### 3.2. Measures and Data Collection

Eudaimonic Well-being. We used the adapted version of Work as Meaning Inventory (WAMI); [64,65], a 10-item measure assessing: search for Greater Good Motivation (3 items; e.g., “My work helps me make sense of the world around me”), Positive meaning (4 items; e.g., “I have found a meaningful career”), and Contribution to meaning-making (3 items; e.g., “I view my work as contributing to my personal growth”). The inventory (M = 4.02; SD = 1.14, Cronbach α = 0.95) was used to assess the overall Meaning of Work, and items were rated from 1 (absolutely untrue) to 5 (absolutely true).

Performance. Performance was assessed via the Short Version of General Self-Assessment Scale of Job Performance (SVSAJP) [66]. Originally developed in a Brazilian–Portuguese version, it is a 10-item measure assessing Task and Context performance (e.g., “I take initiatives to improve my results at work”). The scale (M = 4.18; SD = 0.82; Cronbach α = 0.92) was used to assess general job performance. Items were rated from 1 (absolutely false) to 5 (absolutely true).

Flexibility I-deal. The HR flexibility i-deal was measured using a 4-item scale (HR i-deal [67], adapted to Brazil [68], e.g., “I try to negotiate my job conditions with the company”). The scale is a single factor (M = 3.39; SD = 1.21; Cronbach α = 0.79) rated from 1 (Never) to 5 (Always).

Recovery. The Recovery Experience Questionnaire [11], adapted to Brazil [69], is composed of four types of recovery experience (psychological detachment, relaxation, mastery, and control). Participants indicated on a 5-point scale (1 = never to 5 = always) the extent to which each recovery experience described the psychological detachment (3-item; M = 2.41; SD = 1.23; Cronbach α = 0.75, e.g., “I forget about work”), relaxation (3-item; M = 3.16; SD = 1.22; Cronbach α = 0.88, e.g., “I do relaxing things”), mastery (3-item; M = 3.70; SD = 1.12; Cronbach α = 0.86, e.g., “I learn new things”), and control (3-item; M = 3.47; SD = 1.16; Cronbach α =.86, e.g., “ I decide my own schedule”).

We followed the international ethical recommendations for data collection (We followed the general principles related to human research from the Helsink Declaration. Available online: https://www.wma.net/what-we-do/medical-ethics/declaration-of-helsinki/ (accessed on 23 November 2021)). The research objectives were initially explained, and the respondents’ participation was completely voluntary. The respondents were free to withdraw from the survey at any time if they thought it necessary. In the end, we provided a contact to offer support in case the participant felt uncomfortable. Furthermore, we guaranteed the anonymity of the responses and kept the information received confidential. All data obtained were analyzed in aggregate form so individual responses could not be identified.

### 3.3. Data Analysis

#### 3.3.1. Preliminary Analysis

There were no missing data due to the data collection procedure. We performed normality tests, and variables did not present any problems. The exception was the performance scale, which showed negative skewness, typical for this self-report variable. Before performing the cluster analysis, we conducted a reliability analysis, and all the scales presented adequate rates; the cluster analysis was conducted using z-scores.

K-means cluster analysis. The sample was divided into clusters using the two-step cluster analysis method with the support of the SnowCluster module developed in [70] for the Jamovi Program (version 1.6). Step 1 of the two-step cluster method undertakes a similar procedure to the k-means algorithm. Based on these results, the method carried out a modified hierarchical agglomerative clustering procedure that sequentially combined objects to form homogenous clusters. A visual inspection was performed with plots also conducted in the Jamovi Program but with the Factor Extra package [71]. After analyzing fit information and quality cluster indexes, the sample was classified into groups that reflect different MOW and performance patterns.

#### 3.3.2. Regression Analysis

We conducted multinomial logistic regression with the nnet package [72], where the clusters identified were variable criteria. We consider the recovery experiences as moderators in the relationship between i-deals and the clusters.

## 4. Study 1: Results

A four-cluster solution was identified. Table 1 depicts the size and centroids of each cluster, expressed in standardized scores. Cluster types reflect the relations considering the centroids on the variables (Meaning of Work and Job Performance).

**Figure 5:**
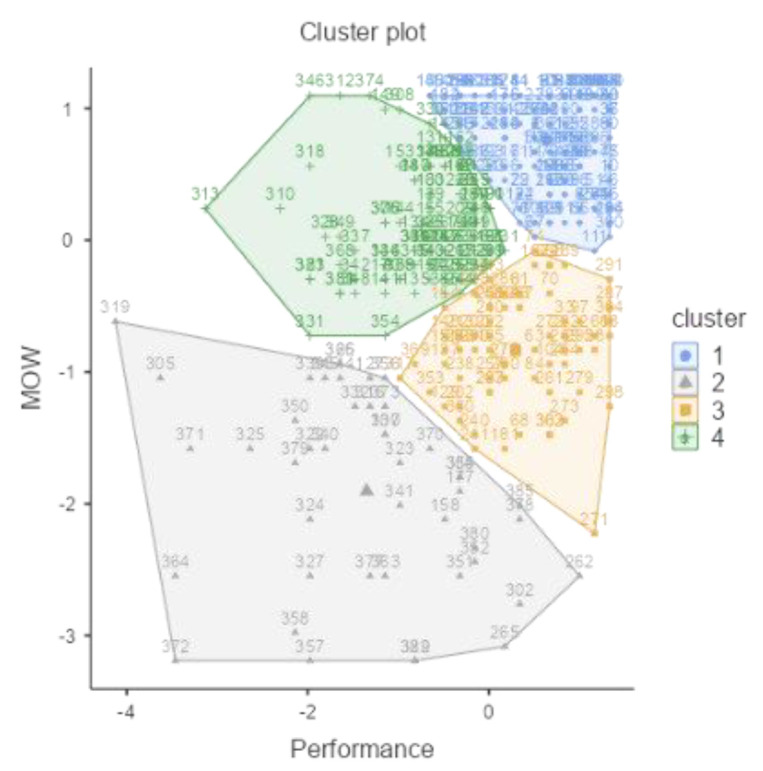
Plot of means across clusters between Job Performance and Meaning of Work. Cluster 1: happy-productive; Cluster 2: Unhappy-productive; Cluster 3: Unhappy-unproductive; Cluster 4: Happy-unproductive. (Study 1; *N* = 386) (r = 0.43; *p* < 0.001).

The sum of the synergic groups, happy-productive (38.34%) and unhappy-unproductive (11.40%), aggregates around 50% of the sample. The sum of the antagonist groups, unhappy-productive (18.13%) and happy-unproductive (32.12%), aggregates the other 50%. Figure 5 represents each cluster.

Figure 5 shows that the smaller cluster (N = 44) is also the one with the largest dispersion. This cluster is the unhappy-unproductive (Cluster 3).

Table 2 describes the multinomial regression, before and with moderation variables. These results do not support H1.

**Figure 6:**
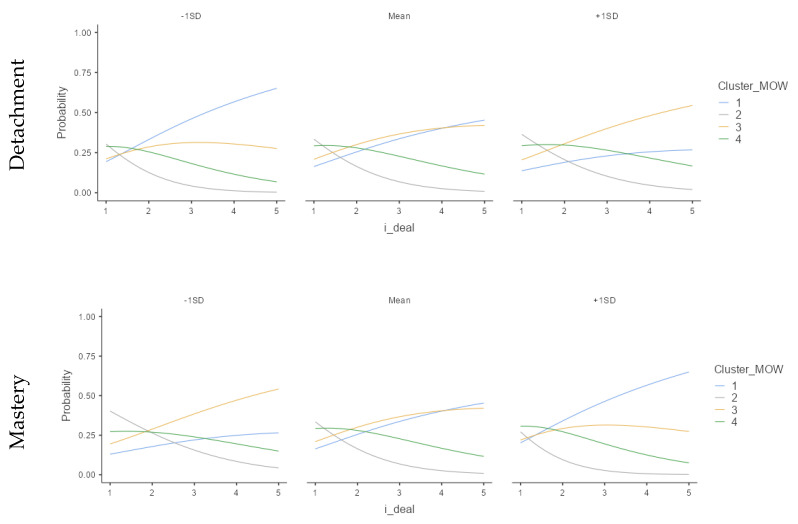

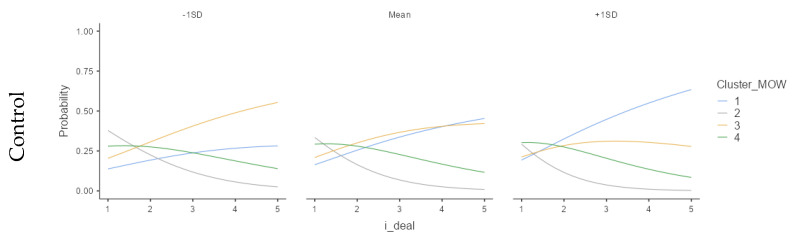
Recovery and Flexibility i-deal interaction related to Meaning of Work and Job Performance clusters. Cluster 1: happy-productive; Cluster 2: unhappy-productive; Cluster 3: unhappy-unproductive; Cluster 4: happy-unproductive. (Study 1; *N* = 386).

According to Model 1, the i-deal only directly predicts the unsustainable cluster (U-U), but only in a marginally significant way. Significant results are also observed in the cluster with a good perception of performance, but not in the antagonistic arrangement, i.e., low perception of work meaning (U-P). However, the relation changes, moderated by recovery experiences, supporting H2 (Figure 6).

Regarding Detachment from work, we found that the more a person uses Detachment and Flexibility i-deal, the more likely they were to be in the unsustainable well-being at work clusters. Meanwhile, Relaxation experience does not moderate the relation. Mastery and Control show similar results aligned with H2, which suggested that the higher the Mastery or Control and Flexibility i-deal, the higher the probability of being in the happy-productive cluster, so it is possible to affirm that H2 is partially supported.

The results particularly challenge the direct relation between Flexibility i-deals and sustainable pattern. One possible explanation for this result is that, at the beginning of the pandemic outbreak, when this data collection was conducted, employees who asked for personal arrangements were the ones that were vulnerable and had personal issues. Additionally, the operationalization used (MOW) was susceptible to the issues already mentioned at that uncertain time.

Moreover, recovery moderates the relation between Flexibility i-deals and sustainable patterns in different ways. When the employee experiences more psychological detachment, at low levels of i-deals, the probability of being on the sustainable pattern increases. However, Mastery and Control improve the relationship between Flexibility i-deals and sustainable patterns, working as a catalytic; in other words, the combination between Flexibility i-deals and Mastery or Control experiences improves the probability of being in a happy and productive pattern. Psychological detachment was already described as a paradoxical variable [12]; the more one needs to detach from work, the less one is able to do it. In this sense, when the worker needs to make i-deals, he or she feels as though they are in debt and cannot detach properly. Another possible explanation is that when a person experienced more psychological detachment and recovered sufficiently, he or she could change the low-i-deal into a challenging demand. The last explanation is that the worker simply does not need to make i-deals.

In summary, we did not find evidence to relate Flexibility i-deals with SWBW directly. Instead, we found that Mastery and Control moderate the relationship between the Flexibility i-deal and the happy and productive pattern. It seems that happy and productive employees experience mastery and control in their leisure time and arrange Flexibility i-deals with their organizations. Hence, happy and productive employees recover by choosing their leisure activities and carrying out challenging leisure experiences. These results align with a study that found that old teachers benefited more from control and mastery during the off-job time [21], but did not support previous research in recovery related to relaxation experience [60].

Ultimately, after one year, the environment changed; vaccination began in Brazil, and we decided to test the same hypothesis adding another operationalization of well-being: positive and low negative emotions. We added emotions based on two criteria. The first was the Gallup report that described Brazil decreasing in positive and increasing in negative emotions; the second was that hedonic well-being is the most used variable tested with performance. 

## 5. Study 2: Examining the Moderating Role of Recovery in the Relationship between i-Deals and Patterns of SWBW to Teleworkers in the Second Brazilian Outbreak

### 5.1. Participants

In the second data collection, 458 participants answered the survey. The inclusion criterion was the same as the first study, i.e., to be teleworking due to the pandemic measures. People that were at home since the first lockdown and recent teleworkers were both included. We applied the same online social network and snowball sampling technique. Again, only people with a formal employment relationship were retained. Study 2 included 281 employees (49.1% fixed employees and 50.9% public servants), 66.9% women. Most were married (55.2% or single 31.0%). In terms of education level, 71.5% had a Master’s degree or superior, and 22.8% had graduated. Of the participants, 52.7% earned more than 10 times the minimum salary in Brazil. Regarding the occupational role, 57% were subordinate, 39% shared roles, and 4% were bosses. 

Regarding experience in teleworking, 75% indicated that they had never worked remotely before the pandemic and 18% answered “sometimes”. Finally, similar to Study 1, most of the participants lived with three other people (28%), followed by two (25%), and one (20%). Additionally, 51% had at least one child living with them.

### 5.2. Measures and Data Collection

Eudaimonic Well-being—MOW. The same WAMI [64,65] was applied. The inventory (M = 3.88; SD = 1.12; Cronbach α = 0.93) was used to assess the overall Meaning of Work.

Hedonic Well-being—Emotions were measured with a short version of the Paschoal and Tamayo [73] scale (English version [74]) based on its factor loading. The scale was used in a unifactorial brief version of the original scale. It was composed of positive emotions (N= 4 items) and a reversed score of negative emotions (5 items) (item of positive emotion example “over the past six months my work made me feel happy”; item of negative emotion example “over the past six months my work made me feel upset”; M = 3.12; SD = 1.08; Cronbach α = 0.85). The response scale was a 5-point agreement scale.

Performance. We used the same SVSAJP measure [66] as Study 1. The scale (M = 4.17; SD = 0.86; Cronbach α = 0.90) was used to assess job performance.

Flexibility I-deal. The same HR flexibility was applied [68]. The scale is a single factor (M = 3.27; SD = 1.27; Cronbach α = 0.76). 

Recovery. The same Recovery Experience Questionnaire [69], composed of four types of recovery experiences (psychological detachment, relaxation, mastery, and control) was applied. It describes psychological detachment (3-item; M = 2.55; SD = 1.18; Cronbach α = 0.81), relaxation (3-item; M = 3.22; SD = 1.11; Cronbach α = 0.83), mastery (3-item; M= 3.43; SD = 1.15; Cronbach α = 0.84), and control (3-item; M = 3.36; SD = 1.13; Cronbach α = 0.86).

The same ethical recommendations for data collection as described for Study 1 were followed. Because we did not expect the restraints to extend for so long, the anonymity guaranteed to the participants made it impossible to conduct a longitudinal study with merged responses. This limitation will be discussed later.

### 5.3. Data Analysis

Preliminary Analysis. We conducted the same steps as in Study 1; no particular problem was found, i.e., there was nothing disturbing the main analysis. We also conducted similar cluster and multinomial regression analyses.

## 6. Study 2: Results

Study 2 took place one year after the first COVID-19 outbreak, in the middle of the second outbreak. We broadened the types of well-being, using two types in this Study: eudaimonic (MOW) and hedonic (emotions). Results are organized describing MOW well-being and after emotions.

### 6.1. Meaning of Work

Another four-cluster solution was identified. Table 3 depicts sizes and standardized centroids. Cluster types reflect the relations of variables (MOW and Job Performance). In general, the groups’ length was similar to that in Study 1: the larger groups in Study 1 were also the larger groups in Study 2. The sum of the synergic groups, happy-productive (38.08%) and unhappy-unproductive (9.61%), aggregates around 50% of the sample. The sum of the antagonist groups, unhappy-productive (21.00%) and happy-unproductive (30.60%), aggregates the other 50%.

**Figure 7:**
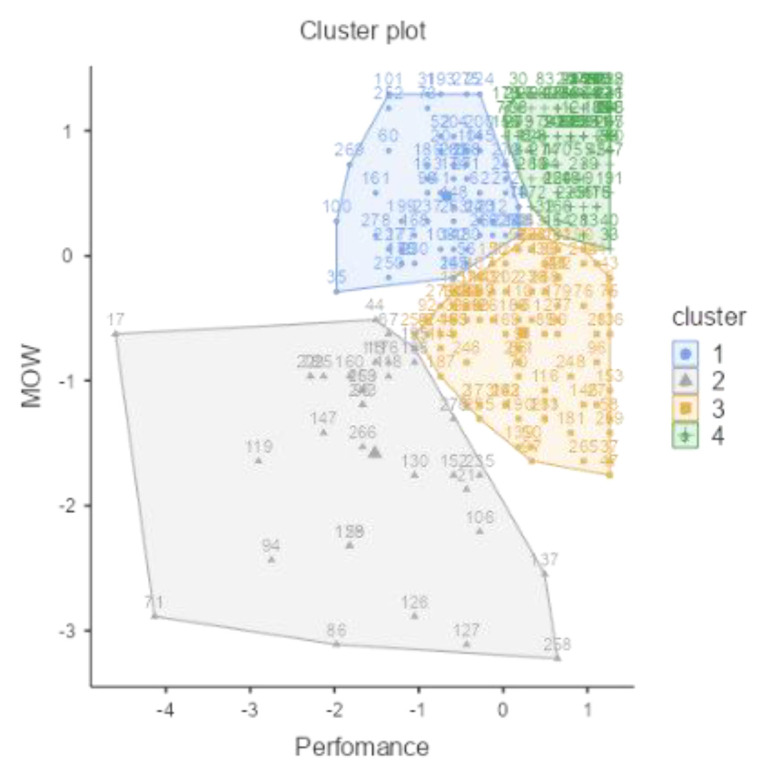
Plot of means across clusters between Meaning of Work and Job Performance. Cluster 1: happy-unproductive; Cluster 2: unhappy-unproductive; Cluster 3: unhappy-productive; Cluster 4: happy-productive. (Study 2; *N* = 281) (r = 0.39; *p* < 0.001).

Once again, the unhappy-unproductive cluster (Cluster 2) was the smallest and most dispersed, and the happy-productive cluster (Cluster 4) was the largest and most concentrated (Figure 7).

Table 4 shows the multinomial moderated regression. For this sample, i-deal predicts a marginally significant eudaimonic sustainable well-being at work pattern (MOW), rejecting H1.

Similar to the previous study, we observed a marginal significance (U-P) or good significance (H-U) for the antagonistic patterns. It is important to highlight that in this case, as opposed to Study 1, the direction of i-deals was as predicted in the hypothesis. Thus, in this year of the COVID-19 pandemic, some results change regarding i-deals. Regarding recovery relationships, Psychological detachment from work and Relaxation were not significant in this sample. Additionally, Mastery and Control experiences moderate the relation between Flexibility i-deals and the patterns, but in the opposite direction; therefore, H2 is not supported.

**Figure 8:**
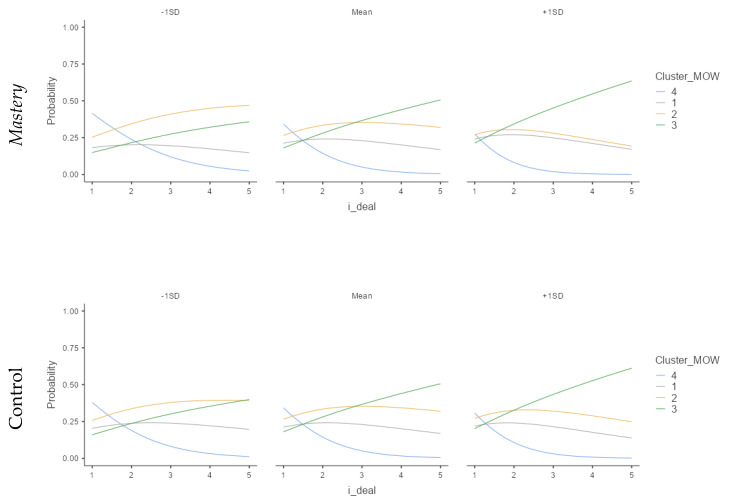
Recovery and Flexibility i-deal interaction related to Meaning of Work and Job Performance clusters. Cluster 1: happy-productive; Cluster 2: Unhappy-productive; Cluster 3: Unhappy-unproductive; Cluster 4: Happy-unproductive. (Study 1; *N* = 281).

The moderated relations, shown in Figure 8, suggest an inverse relationship between Mastery or Control experiences combined with making fewer i-deals and the worker’s happy-productive pattern.

### 6.2. Emotions

Another four-cluster solution was identified. Table 5 depicts the size and standardized centroids of the Emotions and Job Performance cluster. In general, the sizes of the groups were more balanced in terms of length than the Meaning of Work cluster sizes. The sum of the synergic groups, happy-productive (27.05%) and unhappy-unproductive (13.88%), again aggregates around 50% of the sample. The sum of the antagonist groups, unhappy-productive (25.62%) and happy-unproductive (23.49%), aggregates the other 50%.

**Figure 9:**
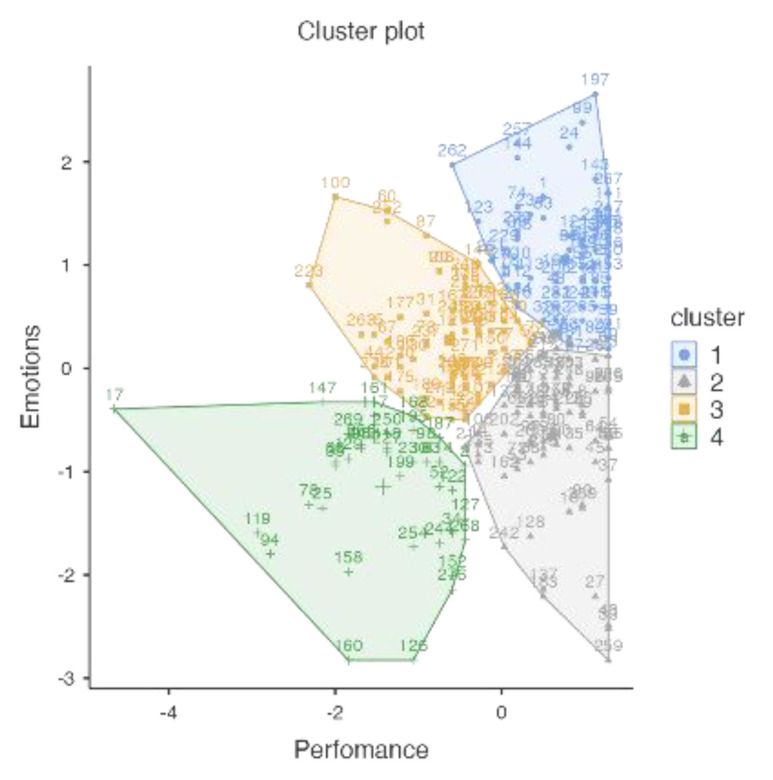
Plot of means across clusters between Emotions and Job Performance. Cluster 1: happy-productive; Cluster 2: unhappy-productive; Cluster 3: happy-unproductive; Cluster 4: unhappy-unproductive. (Study 2; *N* = 281) (r = 0.26; *p* < 0.001).

The hedonic sustainable well-being at work (emotions) shows a different dispersion pattern than the eudaimonic sustainable well-being at work (MOW). The clusters are balanced (Figure 9).

The results described in Table 6 also do not support H1, meaning that Flexibility i-deals do not predict the sustainable pattern directly. Similar to Study 1, with the MOW variable, only a marginally significant relationship was observed with the non-sustainable synergistic pattern (U-U). Moreover, the mediation of recovery experiences showed significance for Detachment, Relaxation, and Mastery experiences (Model 2).

**Figure 10:**
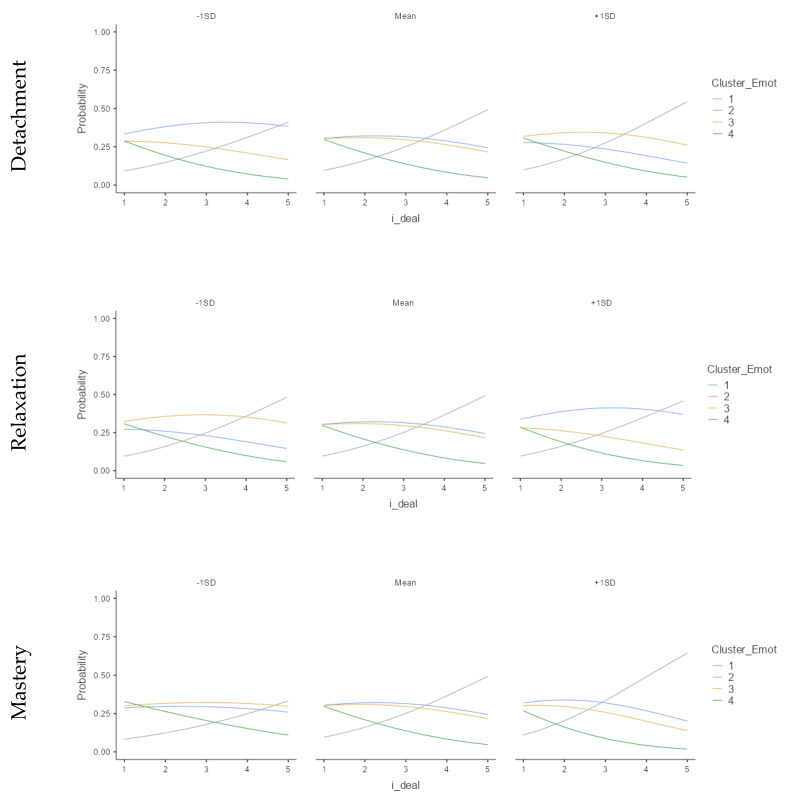
Recovery and Flexibility i-deal interaction related to Emotions and Job Performance clusters. Cluster 1: happy-productive; Cluster 2: Unhappy-productive; Cluster 3: Unhappy-unproductive; Cluster 4: happy-unproductive (Study 1; *N* = 386).

Psychological detachment follows the same pattern as in Study 1; higher detachment relates to a lower probability of being in the sustainable patterns. However, Relaxation and Mastery were the opposite, and higher experiences were related to a higher probability of being on the sustainable pattern, supporting H2. Figure 10 describes the moderation pattern between recovery experiences and i-deals.

Some patterns should be noted. The happy-productive cluster (Cluster 1) decreases when the Detachment recovery experience increases; still, it increases in Relaxation and Mastery experiences. Interestingly, the unhappy-productive cluster (Cluster 2), although not significant, showed a similar pattern on the three experiences and was notably different from the others; in other words, the more the i-deal, the more unhappy-productive.

In summary, Study 2 shows that recovery experiences moderate Flexibility i-deals and SWBW pattern relation. Psychological detachment showed a similar pattern, both in Study 1 and in Study 2. The Psychological detachment experience inversely correlates with the likelihood to be in the sustainable pattern. Relaxation seems to mediate only hedonic variables (i.e., emotions). Additionally, the interaction between Flexibility i-deals and two recovery experiences (Mastery and Control) was peculiar. At the second outbreak, happy-productive workers were more likely to have Flexibility i-deals and less Mastery and Control experiences in their leisure time.

## 7. Discussion

This work assessed the moderating role of recovery from work stress in the relationship between Flexibility i-deals and SWBW patterns in Brazilian teleworkers. This research tested two main hypotheses: (1) that i-deals are directly associated with the happy-productive pattern (H1), and that recovery experiences moderate the relationship between i-deals and the happy-productive pattern, enhancing this relationship (H2). The analysis supports the idea that recovery experiences moderated the relationship between i-deals and sustainable well-being at work patterns, partially confirming our hypothesis (H2). However, recovery experiences moderate this relationship in several ways, depending on the outbreak (Study 1 or 2) and the operationalization used for well-being. In the first and second outbreak, we found that more Psychological detachment was associated with more Flexibility i-deals and unsustainable well-being at work patterns. Relaxation moderates the relationship between Flexibility i-deals and SWBW only for emotions. Finally, Mastery and Control moderated the relationship between Flexibility i-deal and the sustainable pattern catalyzing them in the first pandemic outbreak. However, in the second outbreak, Mastery and Control moderated the relationship in the opposite direction. These results offer several implications for both theory and practice.

### 7.1. Theoretical Implications

This study has at least five theoretical implications. The first explains how teleworkers and organizations managed to shelter Brazil’s stressful environment from the effects of the pandemic [2,3,6]. Some organizations facilitated flexibility i-deals and helped teleworkers face highly stressful situations following the Job Demands and Job Resources model to diminish stress [4,63]. However, this arrangement works differently depending on the situation, rejecting the “one-size-fits-all” idea.

Second, we showed how teleworkers develop individual resources that self-aid their recovery. For instance, upon the scarcity of resources due to the fear of viral infection (e.g., no social contact or going to public places), the use of relaxation-orientation activities through mindfulness [60] helps manage the stressful demands of work and the uncertain environment, which is in line with COR theory [51,58].

Third, this article also offers empirical evidence to the i-deal, SWBW, and recovery literature by expanding our knowledge regarding the direct and moderated effects of organizational and personal resources on sustainable patterns [4,17,18,75,76]. In particular, our results were unexpected because, generally speaking, we found no significant direct effects. Still, it is possible to discuss the directions of the relationships. Study 1 reflects that the beginning of the pandemic (2–3 months into the pandemic in Brazil) was hard, but at the same time, those who could work from home considered their privileges over those who were unable to do it [43,77]. In addition, the uncertainty [3] was more widespread than in Study 2. One year later, the workers were exhausted, and the customized practices of i-deals generated an increase in eudaimonic well-being, perhaps accompanied by a lower fear of losing their jobs. Therefore, i-deals could directly predict the eudaimonic sustainable pattern. It must be pointed out that the reality of vaccines also crossed the relations between workers and employees after this time. Despite the lack of credibility of most Brazilian institutions, the SUS (Single Health System) received a great reception from the Brazilian population. Although we have other reasons that affected labor relations, as in other countries, the vaccine generated hope for change in the pandemic scenario.

Contrary to empirical research that showed that i-deals protect emotional contagion [14], i-deals did not act as a protective mechanism for emotions in this study. One possible explanation for these results is that employees resort to i-deals as a treatment-oriented strategy (when they are already experiencing ill-being) and not a prevention-oriented strategy. In any event, hedonic well-being, understood as emotions, decreased in Brazil last year [2]. We knew that, in general, the Brazilian organizations showed little or no practical support to work from home [43,78]. Thus, people adapted to the situation and, after one year, things changed, hence the result.

Regarding the low i-deal impact in this sample, there is another explanation: *jeitinho*. Some coercive practices and Brazil’s gap between formal bureaucracy and lenient social practices open places for different arrangements [5,8]. *Jeitinho,* a Brazilian way to conduct and solve problems, could blur the effects of i-deals. *Jeitinho* is defined as a “voluntary act that variously uses creativity, deception, interpersonal empathy, and cordiality to solve an unexpected problem or to obtain favors” [79](p. 333). This arrangement is so widespread in the culture that it could be difficult for a Brazilian not to think that the organization wants something in return for i-deals. Thus, Brazilian workers could interpret i-deals as shady [10].

Fourth, this work contributes to the SWBW proposition made by Peiró et al. [16]. They pointed out that linearity, operationalization, predictors, and long-run affect the relationship between well-being and performance. We used two different well-being measures, hedonic and eudaimonic, and found four patterns for both (Figure 3, Figure 5 and Figure 7). Eudaimonic clusters (emotions) were more linear than the hedonic variable. We also obtained four patterns of sustainable well-being at work with self-performance [18]. We added a moderation model between individual arrangements (i-deals) and personal resources, i.e., recovery experience, to predict both hedonic and eudaimonic clusters. This combination is partially aligned with our expectations. Although limitations in the data collection hindered us from testing a lagged model, we could infer some changes in the sample results from the uncertainty of the environment [3] and the actions carried out by workers and organizations. It is crucial to continue measuring these variables because the dynamics between them could change through time, as in the present case. Additionally, the way we measured SWBW from eudaimonic and hedonic measures revealed how MOW (future-oriented eudaimonic happiness) changed through time due to changes in the environment, and how emotions (hedonic happiness) are more influenced by relaxation than other recovery experiences. In this sense, our study covered the two main propositions in the field of happiness and performance [16,26].

Finally, this research deepens our understanding of the mechanisms that moderate situational variables (i-deals) with outcomes (clusters) by revealing the importance of time and context (at the beginning of the pandemic and one year after) to understand the effect of different recovery experiences. Sonnentag [12] suggested that this paradoxical tension can be explained by “high negative activation, depletion of energetic resource, and constant connectivity to work caused by job stressors as well as individual and organizational factors” (p. 175). Sonnentag also describes, in her recovery paradox proposition, that those who have a high need for recovery have a low propensity to actually recover, particularly to detach from work. In our study, we observed the mechanisms described by this author, particularly in the eudaimonic measure at the first pandemic outbreak and the hedonic measure in the second, opening a new avenue of research.

It is important to remark that Mastery and Control recovery experience changed direction in this period. In Study 1, they protected and increased the probability of being in the happy-productive pattern. However, in Study 2, MOW clusters showed the opposite. In contrast, emotions did not change between Studies. We could interpret that one year of the COVID-19 pandemic increased the quantitative and qualitative demands, and even Mastery and Control drained in the long run, harming exhausted people who needed to protect their scarce resources [51,58,80]; this interpretation is aligned with the COR theory [51]. He relaxation experience, which shows no effect on the Meaning of Work clusters, reveals an essential and protective experience for positive emotions, replicating previous results [60]. These results provide a new nuance of the recovery experience repercussions.

### 7.2. Managerial Implications

Our findings have implications for human resource management and individuals, especially for the employee-support process regarding i-deals and recovery experiences. For i-deals, HR practitioners should consider how, when, and what kind of i-deal to conduct and what kind of outcome to expect. Within an increasing workplace diversity, i-deals should facilitate employees to deal with their needs [20]. However, because workers or their colleagues might think of i-deals as shady, their success strongly depends on interpretation and context [10]. Flexibility i-deals are tools to increase the employees’ resources and help them manage stress. If managers arrange i-deals with employees, they could perform better and be happier, provided employees combine them with recovery experiences.

Another managerial implication is related to the recovery process. Our results showed the benefits of the recovery experiences during off-job time as a moderator of organizational arrangements. These results support the positive interaction between work and non-work factors and open avenues for designing interventions to enrich this interaction [81,82]. However, different recovery experiences suggest different outcomes depending on the situation. Relaxation, Mastery, and Control experiences showed positive results regarding well-being and performance. However, Mastery and Control only showed positive results at the beginning of the pandemic, probably because people still had resources to apply in other life domains [19,83]. In the long run, the depletion of resources seems to backfire, changing these results [51]. In this sense, it is essential to know the employee’s situation and context before suggesting one or another experience.

### 7.3. Limitations and Future Research Suggestions

This study described some interesting results; however, it was restricted by some limitations. The first and foremost limitation was that the sampling method employed non-longitudinal data collection. At the beginning of the sampling, we did not expect such a long pandemic. Because we used the same snowball technique with colleagues, friends, and family, we probably collected data from the same public, but we could not merge it. Future longitudinal studies in countries or organizations that suffer contextual effects [55], with or without a pandemic environment, should test long- and short-term responses and the extent and conditions where the recovery experiences protect the teleworkers. In addition, it is crucial to consider that half of the labor market in Brazil is informal; future studies should focus on this situation [77].

Aside from the common method bias, which implies that the results should be interpreted cautiously, the results are consistent with previous studies. The inability to assess outcomes at other sources limits the extension of the results. Future research should explore the mechanisms behind the relation between work arrangements, outcomes, and personal resources through the lens of more than one source [43,57]. Recent studies on telecommuting as a function of COVID-19 have shown interesting relationships between perceived job characteristics and procrastination and how both predict well-being and performance [84]. Future studies should also analyze the work characteristics among teleworking i-deals initiated by the employee or forced by the organization.

Our study showed that no single resource is applicable for employees, and can be detrimental to employees in some circumstances, as pointed out elsewhere [85]. It can mean an inflection point, where increasing the level of resource, recovery and/or i-deal, may even be harmful. Although we showed evidence of the complex relationship between personal and organizational resources [80], we did not include and compared cultural variables [5].

Finally, teleworking has involved a complex modification of the organization of work. This modification could influence many aspects of the health of employees; among these are leadership style (intrusive leadership style), off-time work, and workaholism [86], so these aspects could be analyzed in future studies. In addition, future research should also focus on the relationships between supervisors’ behaviors that facilitate scheduled-flexibility i-deals and employee outcomes [87], including the organization’s perspective and the benefits it derives from telecommuting work arrangements.

## 8. Conclusions

This study presented a complex insight into recovery experiences and the relationship between flexibility i-deals and SWBW. Important factors that may lead to recovery and the establishment of i-deals include the uncertainty of the Brazilian environment and the duration of the pandemic (both short term and long term). Experiences from the first outbreak changed after one year, and some of them, i.e., Mastery and Control, seem to have had a backfiring effect on MOW when resources were scarcer. However, the Relaxation that showed no effect on MOW presented a positive effect on emotions. By highlighting these relationships, we provide insights into the relations of eudaimonic and hedonic patterns considering Brazil’s current work environment, recovery experiences in the short and long term, and SWBW patterns.

## Figures and Tables

**Table 1:** Four-cluster solution, cluster standardized means and group-sizes, Meaning of Work and Job Performance (Study 1; *N* = 386).

Cluster	Type	*N*	Centroids	
			Performance	Meaning of Work
1	Happy-productive	148	0.860	0.675
2	Unhappy-productive	70	0.225	−1.178
3	Unhappy-unproductive	44	−1.855	−1.385
4	Happy-unproductive	124	−0.496	0.351

**Table 2:** Regression coefficients of i-deal moderated by recovery on Meaning of Work and Job Performance clusters (Study 1; *N* = 386).

Variable	2 (U-P)	4 (H-U)	3 (U-U)
*Model 1*			
Flexibility i-deal	1.03 **	0.35	0.50 ^+^
*Model 2*			
Flexibility i-deal * Detachment	0.26 ***	0.18 ***	0.15 **
Flexibility i-deal * Relaxation	−0.07	−0.07	0.05
Flexibility i-deal * Mastery	−0.43 ***	−0.16 **	−0.16 ***
Flexibility i-deal * Control	−0.30 ***	−0.13 **	−0.15 ***

McFedder Model 1 = 0.04; Model 2 = 0.15; χ2 = 96.2; DF = 12; U-P = Unhappy-Productive; H-U = Happy-Unproductive; U-U = Unhappy-Unproductive; ^+^ *p* < 0.1 * *p* < 0.05; ** *p* < 0.01; *** *p* < 0.001.

**Table 3:** Four-cluster solution, cluster standardized means, and group-sizes between Meaning of Work and Job Performance (Study 2; *N* = 281).

Cluster	Type	Frequency	Centroids	
			Performance	Meaning of Work
1	Happy-unproductive	86	−0.625	0.159
2	Unhappy-unproductive	27	−1.963	−1.375
3	Unhappy-productive	59	0.436	−1.057
4	Happy-productive	107	0.775	0.803

**Table 4:** Regression coefficients of flexibility i-deal moderated by recovery on Meaning of Work and Job Performance clusters (Study 2; *N* = 281).

Variable	3 (U-P)	1 (H-U)	2 (U-U)
*Model 1*			
Flexibility i-deal	−0.74 ^+^	1.03 *	−0.37
*Model 2*			
Flexibility i-deal * Detachment	−0.03	−0.16	0.02
Flexibility i-deal * Relaxation	0.04	0.00	0.06
Flexibility i-deal * Mastery	0.37 ***	0.33 **	0.23
Flexibility i-deal * Control	0.23 **	−0.15	0.14

McFedder Model 1 = 0.04; Model 2 = 0.11; χ^2^ = 53,6; DF = 12; U-P = Unhappy-productive; H-U = happy-unproductive; U-U = unhappy-unproductive; ^+^ *p* <.1; * *p* < 0.05; ** *p* < 0.01; *** *p* < 0.001; *N* = 281.

**Table 5:** Four-cluster solution, cluster standardized means and group-sizes for Emotions and Job Performance (Study 2; *N* = 281).

Cluster	Type	Frequency	Centroids	
			Performance	Emotions
1	Happy-productive	76	0.752	1.000
2	Unhappy-productive	72	0.592	−0.683
3	Happy-unproductive	66	−0.670	0.273
4	Unhappy-unproductive	39	−1.426	−1.150

**Table 6:** Regression coefficients of Flexibility i-deal moderated by recovery on Emotions and Job Performance clusters (Study 2; *N* = 281).

Variable	2 (U-P)	3 (H-U)	4 (U-U)
*Model 1*			
Flexibility i-deal	−0.17	0.25	0.65 ^+^
*Model 2*			
Flexibility i-deal * Detachment	0.13 *	0.14 *	0.12
Flexibility i-deal * Relaxation	−0.10	−0.19 **	−0.15 ^+^
Flexibility i-deal * Mastery	0.09	−0.05	−0.15 *
Flexibility i-deal * Control	0.10^+^	0.04	−0.11

McFedder Model 1 = 0.04 Model 2 = 0.10 DF = 12; U-P = Unhappy-productive; H-U = happy-unproductive; U-U = unhappy-unproductive; ^+^ *p* < 0.1; * *p* < 0.05; ** *p* < 0.01.

## Data Availability

Study 1, data is available at: Queiroga, F.; Pérez-Nebra, A. (2021), “COVID-19 BR T1”, Mendeley Data, V2, doi: 10.17632/8xbrd3g4by.2 (accessed on 10 September 2021) Study 2, the data is available at: Queiroga, F.; Pérez-Nebra, A. (2021), “COVID-19 BR T2”, Mendeley Data, V2, doi:10.17632/4cbwt33w4s.2 (accessed on 21 September 2021).

## References

[1] Johns Hopkins University & Medicine Coronavirus Resource Center. https://coronavirus.jhu.edu/map.html.

[2] Helliwell J., Layard R., Sachs J.D., De Neve J.-E., Aknin L., Wang S. (2021). World Happiness Report 2021.

[3] Milliken F.J. (1987). Three Types of Perceived Uncertainty About the Environment: State, Effect, and Response Uncertainty. Acad. Manag. Rev..

[4] Karasek R. (1979). Job Demands, Job Decision Latitude, and Mental Strain: Implications for Job Redesign. Adm. Sci. Q..

[5] Gelfand M.J., Jackson J.C., Pan X., Nau D., Pieper D., Denison E., Dagher M., Van Lange P.A.M., Chiu C.-Y., Wang M. (2021). The Relationship between Cultural Tightness–Looseness and COVID-19 Cases and Deaths: A Global Analysis. Lancet Planet Health.

[6] The Lancet (2020). COVID-19 in Brazil: “So What?”. Lancet.

[7] Beugelsdijk S., Welzel C. (2018). Dimensions and Dynamics of National Culture: Synthesizing Hofstede With Inglehart. J. Cross Cult. Psychol..

[8] Gelfand M.J., Raver J.L., Nishii L., Leslie L.M., Lun J., Lim B.C., Duan L., Almaliach A., Ang S., Arnadottir J. (2011). Differences Between Tight and Loose Cultures: A 33-Nation Study. Science.

[9] Gelfand M.J., Nishii L.H., Raver J.L. (2006). On the Nature and Importance of Cultural Tightness-Looseness. J. Appl. Psychol..

[10] Rousseau D.M. (2005). I-Deals: Idiosyncratic Deals Employees Bargain for Themselves.

[11] Sonnentag S., Fritz C. (2007). The Recovery Experience Questionnaire: Development and Validation of a Measure for Assessing Recuperation and Unwinding From Work. J. Occup. Health Psychol..

[12] Sonnentag S. (2018). The Recovery Paradox: Portraying the Complex Interplay between Job Stressors, Lack of Recovery, and Poor Well-Being. Res. Organ. Behav..

[13] De Jonge J. (2020). What Makes a Good Work Break? Off-Job and on-Job Recovery as Predictors of Employee Health. Ind. Health.

[14] Bal P.M., Boehm S.A. (2019). How Do I-Deals Influence Client Satisfaction? The Role of Exhaustion, Collective Commitment, and Age Diversity. J. Manag..

[15] Hornung S., Rousseau D.M., Glaser J., Lai L., Rousseau D.M., Chang K.T.T., Hornung S., Kim T.G., Anand S., Vidyarthi P.R. (2010). Good Citizens In Poor-Quality Relationships: Idiosyncratic Deals as a Substitute For Relationship Quality University of Illinois at Chicago University of Illinois at Chicago. Acad. Manag. J..

[16] Peiró J.M., Ayala Y., Tordera N., Lorente L., Rodríguez I. (2014). Sustainable Well-Being at Work: A Review and Reformulation. Pap. Del Psicólogo.

[17] Peiró J.M., Tordera N., Lorente L., Rodríguez I., Ayala Y., Latorre F. (2015). Bienestar Sostenible En El Trabajo. Conceptualización, Antecedentes y Retos, Psyciencia. Rev. Latinoam. Cienc. Psicológica.

[18] Peiró J.M., Kozusznik M., Molina I.R., Tordera N. (2019). The Happy-Productive Worker Model and beyond: Patterns of Wellbeing and Performance At work. Int. J. Environ. Res. Public Health.

[19] Kelly C.M., Rofcanin Y., Las Heras M., Ogbonnaya C., Marescaux E., Bosch M.J. (2020). Seeking an “i-Deal” Balance: Schedule-Flexibility i-Deals as Mediating Mechanisms between Supervisor Emotional Support and Employee Work and Home Performance. J. Vocat. Behav..

[20] Bal M., Rousseau D.M. (2015). Idiosyncratic Deals between Employees and Organizations Conceptual Issues, Applications and the Role of Co-Workers.

[21] Virtanen A., De Bloom J., Kinnunen U. (2020). Relationships between Recovery Experiences and Well-Being among Younger and Older Teachers. Int. Arch. Occup. Environ. Health.

[22] Fritz C., Sonnentag S. (2005). Recovery, Health, and Job Performance: Effects of Weekend Experiences. J. Occup. Health Psychol..

[23] Karasek R., Baker D., Marxer F., Ahlbom A., Theorell T. (1981). Job Decision Latitude, Job Demands, and Cardiovascular Disease: A Prospective Study of Swedish Men. Am. J. Public Health.

[24] Peccei R., van de Voorde K., van Veldhoven M., Paauwe J., Guest D., Wright P. (2013). HRM, Well-Being and Performance: A Theoretical and Empirical Review. HRM & Performance.

[25] Wright T.A., Cropanzano R., Bonett D.G. (2007). The Moderating Role of Employee Positive Well Being on the Relation between Job Satisfaction and Job Performance. J. Occup. Health Psychol..

[26] Peiró J.M., Montesa D., Soriano A., Kozusznik M.W., Villajos E., Magdaleno J., Djourova N.P., Ayala Y. (2021). Revisiting the Happy-Productive Worker Thesis from a Eudaimonic Perspective: A Systematic Review. Sustainability.

[27] Meijman T.F., Mulder G., Drenth P.J.D., Thierry H., de Wolff C.J. (1998). Psychological Aspects of Workload. Handbook of Work and Organizational Psychology.

[28] Pratt M., Ashforth B., Cameron K.S., Dutton J.E., Quinn R.E. (2003). Fostering Meaningfulness in Working and at Work. Positive Organizational Scholarship: Foundations of a New Discipline.

[29] Ryan R.M., Deci E.L. (2001). On Happiness and Human Potentials: A Review of Research on Hedonic and Eudaimonic Well-Being. Annu. Rev. Psychol..

[30] Warr P. (1990). The Measurement of Well-Being and Other Aspects of Mental Health. J. Occup. Psychol..

[31] Diener E., Suh E.M., Lucas R.E., Smith H.L. (1999). Subjective Well-Being: Three Decades of Progress. Psychol. Bull..

[32] Wigen B., Barret H. Performance Management Must Evolve to Survive COVID-19. https://www.gallup.com/workplace/318029/performance-management-evolve-survive-covid.aspx?thank-you-subscription-form=1.

[33] Tsukamoto Y. (2021). Rethinking Telecommuting with an I-Deals Perspective. Ann. Bus. Adm. Sci..

[34] Ornell F., Halpern S.C., Kessler F.H.P., de Magalhães Narvaez J.C. (2020). The Impact of the COVID-19 Pandemic on the Mental Health of Healthcare Professionals. Cad. Saude Public..

[35] Liao C., Wayne S.J., Rousseau D.M. (2016). Idiosyncratic Deals in Contemporary Organizations: A Qualitative and Meta-Analytical Review. J. Organ. Behav..

[36] Hornung S., Rousseau D.M., Glaser J. (2008). Creating Flexible Work Arrangements through Idiosyncratic Deals. J. Appl. Psychol..

[37] Cropanzano R., Anthony E.L., Daniels S.R., Hall A.V. (2017). Social Exchange Theory: A Critical Review with Theoretical Remedies. Acad. Manag. Ann..

[38] Singh S., Vidyarthi P.R. (2018). Idiosyncratic Deals to Employee Outcomes: Mediating Role of Social Exchange Relationships. J. Leadersh. Organ. Stud..

[39] Hornung S., Rousseau D.M., Glaser J. (2009). Why Supervisors Make Idiosyncratic Deals: Antecedents and Outcomes of i-Deals from a Managerial Perspective. J. Manag. Psychol..

[40] Rosen C.C., Slater D.J., Chang C.-H., Johnson R.E. (2013). Let’s Make a Deal. J. Manag..

[41] Davis A.S., Van der Heijden B.I.J.M. (2018). Reciprocity Matters: Idiosyncratic Deals to Shape the Psychological Contract and Foster Employee Engagement in Times of Austerity. Hum. Resour. Dev. Q..

[42] Guerrero S., Bentein K., Garcia-Falières A. (2021). Countering the Effects of Occupational Stigma on Emotional Exhaustion and Absences with Idiosyncratic Deals. Int. J. Stress Manag..

[43] Mishima-Santos V., Sticca M.G., Pérez-Nebra A.R. (2021). Wellbeing and Work Design in Brazilian Teleworkers. Front. Psychol..

[44] Bal P.M., Vossaert L. (2019). Development of an I-Deals Motivation and Management Measure. J. Pers. Psychol..

[45] Liu J., Lee C., Hui C., Kwan H.K., Wu L.-Z. (2013). Idiosyncratic Deals and Employee Outcomes: The Mediating Roles of Social Exchange and Self-Enhancement and the Moderating Role of Individualism. J. Appl. Psychol..

[46] Quinones C., Rodríguez-Carvajal R., Griffiths M.D. (2017). Testing a Eustress–Distress Emotion Regulation Model in British and Spanish Front-Line Employees. Int. J. Stress Manag..

[47] Van Dam A., Noordzij G., Born M. (2021). Social Workers and Recovery from Stress. J. Soc. Work.

[48] Guo L.N., Zhao R.L., Ren A.H., Niu L.X., Zhang Y.L. (2020). Stress Recovery of Campus Street Trees as Visual Stimuli on Graduate Students in Autumn. Int. J. Environ. Res. Public Health.

[49] Mohd Fauzi M.F., Mohd Yusoff H., Muhamad Robat R., Mat Saruan N.A., Ismail K.I., Mohd Haris A.F. (2020). Doctors’ Mental Health in the Midst of COVID-19 Pandemic: The Roles of Work Demands and Recovery Experiences. Int. J. Environ. Res. Public Health.

[50] Lira P., Pérez-Nebra A.R., Queiroga F. (2021). Modelo Integrado de Burnout Entre Policiais Do Distrito Federal: Uma Ampliação Conceitual. Psicol. Organ. Trab..

[51] Hobfoll S.E. (1998). Stress, Culture, and Community: The Psychology and Physiology of Stress.

[52] Grandey A.A., Sayre G.M., French K.A. (2021). “A Blessing and a Curse”: Work Loss during Coronavirus Lockdown on Short-Term Health Changes via Threat and Recovery. J. Occup. Health Psychol..

[53] Ménard J., Foucreault A., Leduc H., Meunier S., Trépanier S.-G. (2021). A Diary Study on When and With Whom Recovery Experiences Modulate Daily Stress and Worry During a COVID-19 Lockdown. Front. Psychol..

[54] WHO Coronavirus (COVID-19) Dashboard. https://covid19.who.int/.

[55] Demenech L.M., de Carvalho Dumith S., Vieira M.E.C.D., Neiva-Silva L. (2020). Income Inequality and Risk of Infection and Death by Covid-19 in Brazil. Rev. Bras. Epidemiol..

[56] Ritchie H., Mathieu E., Rodés-Guirao L., Appel C., Giattino C., Ortiz-Ospina E., Hasell J., MacDonald B., Beltekian D., Roser M. Brazil: Coronavirus Pandemic Country Profile-Our World in Data. https://ourworldindata.org/coronavirus/country/brazil#what-is-the-daily-number-of-confirmed-deaths.

[57] Faro A., de Andrade Bahiano M., de Cassia Nakano T., Reis C., da Silva B.F.P., Vitti L.S. (2020). COVID-19 and Mental Health: The Emergence of Care. Estud. Psicol..

[58] Hobfoll S.E. (2002). Social and Psychological Resources and Adaptation. Rev. Gen. Psychol..

[59] Maslach C., Schaufeli W.B., Leiter M.P. (2001). Job Burnout. Annu. Rev. Psychol..

[60] Díaz-Silveira C., Alcover C.M., Burgos F., Marcos A., Santed M.A. (2020). Mindfulness versus Physical Exercise: Effects of Two Recovery Strategies on Mental Health, Stress and Immunoglobulin a during Lunch Breaks. a Randomized Controlled Trial. Int. J. Environ. Res. Public Health.

[61] Park Y., Kim S. (2019). Customer Mistreatment Harms Nightly Sleep and Next-Morning Recovery: Job Control and Recovery Self-Efficacy as Cross-Level Moderators. J. Occup. Health Psychol..

[62] Ten Brummelhuis L.L., Bakker A.B. (2012). A Resource Perspective on the Work–Home Interface: The Work–Home Resources Model. Am. Psychol..

[63] Schaufeli W.B., Bakker A.B. (2004). Job Demands, Job Resources, and Their Relationship with Burnout and Engagement: A Multi-Sample Study. J. Organ. Behav..

[64] Leonardo M.d.G.L., Pereira M., Valentini F., Freitas C., Damásio B. (2019). Adaptação Do Inventário de Sentido Do Trabalho (WAMI) Para o Contexto Brasileiro. Rev. Bras. Orientação Prof..

[65] Steger M.F., Dik B.J., Duffy R.D. (2012). Measuring Meaningful Work: The Work and Meaning Inventory (WAMI). J. Career Assess..

[66] De Azevedo Andrade É.G.S., Queiroga F., Valentini F. (2020). Short Version of Self-Assessment Scale of Job Performance. An. Psicol..

[67] Knering A., Tordera N., Villajos E., Latorre F., Pérez-Nebra A. (2019). Individual and group level antecedents in the development of idiosyncratic deals. A cross-level study. Psychologica.

[68] Pérez-Nebra A.R., Latorre F., Tordera N., Lorente L. (2018). Desarrollo de Un Instrumento Para La Medición Del Contrato Idiosincrático: Un Estudio Transcultural. III Congreso Internacional de la Sociedad Científica Española de Psicología.

[69] Pérez-Nebra A.R., Pedersoli M., Rodrigues A., Rodrigues C.M.L., Queiroga F. (2021). Evidence of Validity of the Brazilian-Portuguese Recovery Experience Questionnaire.

[70] Seol H. Snow Cluster Analysis. https://github.com/hyunsooseol/snowCluster.

[71] Kassambara A., Mundt F. factoextra: Extract and Visualize the Results of Multivariate Data Analyses. https://cran.r-project.org/package=factoextra.

[72] Ripley B., Venables W. nnet: Feed-Forward Neural Networks and Multinomial Log-Linear Models. http://www.stats.ox.ac.uk/pub/MASS4/.

[73] Paschoal T., Tamayo Á. (2008). Construção e Validação Da Escala de Bem-Estar No Trabalho. Avaliação Psicológica.

[74] Demo G., Paschoal T. (2016). Well-Being at Work Scale: Exploratory and Confirmatory Validation in the USA. Paidéia.

[75] Karasek R., Theorell T. (1990). Healthy Work: Stress, Productivity, and the Reconstruction of Working Life.

[76] Petrou P., Demerouti E., Schaufeli W.B. (2015). Job Crafting in Changing Organizations: Antecedents and Implications for Exhaustion and Performance. J. Occup. Health Psychol..

[77] Pérez-Nebra A.R., Sklaveniti C., Islam G., Pickett J., Alija M., Bal P.M., Tekeste M., Bazana S., Sanderson Z. (2021). COVID-19 and the Future of Work and Organisational Psychology. SA J. Ind. Psychol..

[78] Lemos A.H.D.C., Barbosa A.D.O., Monzato P.P. (2020). Women in Home Office During the Covid-A9 Pandemic and the Work-Family Conflict Configurations. RAE Rev. Adm. Empres..

[79] Ferreira M.C., Fischer R., Porto J.B., Pilati R., Milfont T.L. (2012). Unraveling the Mystery of Brazilian Jeitinho. Personal. Soc. Psychol. Bull..

[80] Bakker A.B., Demerouti E. (2017). Job Demands-Resources Theory: Taking Stock and Looking Forward. J. Occup. Health Psychol..

[81] Daniel S., Sonnentag S. (2014). Work to Non-Work Enrichment: The Mediating Roles of Positive Affect and Positive Work Reflection. Work Stress.

[82] Karabinski T., Haun V.C., Nübold A., Wendsche J., Wegge J. (2021). Interventions for Improving Psychological Detachment from Work: A Meta-Analysis. J. Occup. Health Psychol..

[83] Bakker A.B., Demerouti E., Grzywacs J., Demerouti E. (2013). The Spillover–Crossover Model. New Frontiers in Work and Family Research.

[84] Wang B., Liu Y., Qian J., Parker S.K. (2021). Achieving Effective Remote Working During the COVID-19 Pandemic: A Work Design Perspective. Appl. Psychol..

[85] Van Veldhoven M., Van den Broeck A., Daniels K., Bakker A.B., Tavares S.M., Ogbonnaya C. (2020). Challenging the Universality of Job Resources: Why, When, and For Whom Are They Beneficial?. Appl. Psychol..

[86] Magnavita N., Tripepi G., Chiorri C. (2021). Telecommuting, off-Time Work, and Intrusive Leadership in Workers’ Well-Being. Int. J. Environ. Res. Public Health.

[87] Taser D., Rofcanin Y., Las Heras M., Bosch M.J. (2021). Flexibility I-deals and prosocial motives: A trickle-down perspective. Int. J. Hum. Resour. Manag..

